# The matricellular protein CCN6 (WISP3) decreases Notch1 and suppresses breast cancer initiating cells

**DOI:** 10.18632/oncotarget.7734

**Published:** 2016-02-25

**Authors:** Wei Huang, Emily E. Martin, Boris Burman, Maria E. Gonzalez, Celina G. Kleer

**Affiliations:** ^1^ Department of Pathology and Comprehensive Cancer Center, University of Michigan, Ann Arbor, MI, USA

**Keywords:** breast cancer, triple negative, CCN6, WISP3, epithelial to mesenchymal transition

## Abstract

Increasing evidence supports that the epithelial to mesenchymal transition (EMT) in breast cancer cells generates tumor initiating cells (TICs) but the contribution of the tumor microenvironment to these programs needs further elucidation. CCN6 (WISP3) is a secreted matrix-associated protein (36.9 kDa) of the CCN family (named after CTGF, Cyr61 and Nov) that is reduced or lost in invasive carcinomas of the breast with lymph node metastasis and in inflammatory breast cancer. CCN6 exerts breast cancer growth and invasion inhibitory functions, but the mechanisms remain to be defined. In the present study we discovered that ectopic CCN6 overexpression in triple negative (TN) breast cancer cells and in cells derived from patients is sufficient to induce a mesenchymal to epithelial transition (MET) and to reduce TICs. *In vivo,* CCN6 overexpression in the TIC population of MDA-MB-231 cells delayed tumor initiation, reduced tumor volume, and inhibited the development of metastasis. Our studies reveal a novel CCN6/Slug signaling axis that regulates Notch1 signaling activation, epithelial cell phenotype and breast TICs, which requires the conserved thrombospondin type 1 (TSP1) motif of CCN6. The relevance of these data to human breast cancer is highlighted by the finding that CCN6 protein levels are inversely correlated with Notch1 intracellular activated form (NICD1) in 69.5% of invasive breast carcinomas. These results demonstrate that CCN6 regulates epithelial and mesenchymal states transition and TIC programs, and pinpoint one responsible mechanism.

## INTRODUCTION

Extracellular matrix (ECM) proteins play an important role in breast cancer initiation and progression [1-3]. Beyond serving as a scaffold for the organization of breast ducts and lobules, the ECM is also a multifunctional regulator of cell behavior [1]. Matricellular proteins comprise a subset of dynamically expressed ECM proteins that exert regulatory rather than structural roles in normal tissues, and are deregulated in cancer [4-8]. Matricellular CCN proteins (named after Cyr61, CTGF, and NOV) are conserved ECM-associated proteins with developmental functions and roles in tumorigenesis [9-15]. Our laboratory has reported that CCN6 is a secreted protein expressed in normal breast epithelium and is reduced or lost in 60% of invasive breast carcinomas and in 79% of inflammatory breast cancers, the most lethal form of locally advanced breast cancer [10, 12, 16]. The high frequency of reduction or loss of CCN6 in biologically aggressive breast cancer suggests a potential role in breast cancer initiation and/or progression.

The epithelial to mesenchymal transition (EMT) has been shown to promote tumor progression in breast and other malignancies [17-20]. Upregulation of EMT transcription factors (EMT-TFs) is sufficient to induce breast tumor initiating cells (TICs) responsible for metastatic dissemination and clinical relapse [20-22]. Our laboratory has reported that CCN6 knockdown in non-tumorigenic breast cells triggers an EMT and invasion [10, 23, 24]. However, whether CCN6 regulates stem cells and the underlying mechanisms are unknown. Further, no studies have been carried out to elucidate the roles of the conserved CCN6 domains in these processes.

In the present study we demonstrate that ectopic CCN6 overexpression in MDA-MB-231 and MDA-MB-436 aggressive TN breast cancer cells induces a mesenchymal to epithelial transition (MET), reduces cancer cell migration and invasion, and is sufficient to decrease TICs, tumor initiation, and metastasis. Mechanistically, our data show that CCN6-mediated MET and reduction in TICs requires the conserved TSP1 motif of the CCN6 protein, and is mediated through inhibition of a novel Slug/Notch1 signaling axis.

## RESULTS

### Ectopic CCN6 overexpression induces a mesenchymal-to-epithelial transition (MET) and decreases breast cancer cell migration

**Figure 1:**
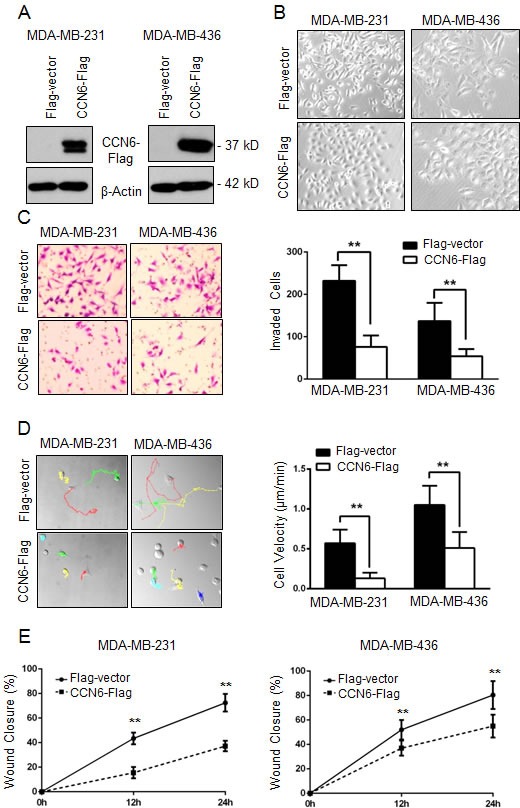
CCN6 overexpression induces phenotypic changes towards mesenchymal to epithelial transition (MET) and decreases breast cancer cell invasion and motility **A.** Western blot analysis of CCN6 protein level on breast cancer cells MDA-MB-231 and MDA-MB-436 following lentiviral transduction with CCN6-Flag or Flag-Vector control. β-Actin was used as loading control. **B.** Representative phase contrast images show that CCN6 overexpression leads to a morphological change from mesenchymal-like to epithelial when compared to controls (200X magnification). **C.** CCN6 overexpression reduces invasion of MDA-MB-231 and -436 cells compared to controls using a reconstituted Boyden basement membrane invasion chamber assay. *Left,* representative images of stained chambers. *Right,* average number of invaded cells of each cell line ± SD. **D.** *Left,* representative images displaying MTrackJ individual MDA-MB-231 and -436 cell tracks, colored dots and connecting lines, from 24 hour time-lapse videos of CCN6-Flag or Flag-Vector cells. Each dot represents a 10 minute time span and closely spaced dots indicate less movement over the elapsed time *versus* widely spaced dots. *Right*, bar graphs show that CCN6 overexpressing cells are significantly slower than controls as demonstrated by the average cell velocity ± SEM (Student's *t*-test, *p < 1×10^−5^, *n* ≥ 25 cells per condition). **E.** Wound healing assays demonstrate that CCN6 overexpressing cells exhibit reduced migration compared to controls. For all experiments, Data are representative of 3 independent experiments. * *p* < 0.05 ** *p* < 0.001.

Breast carcinomas with reduced CCN6 expression have aggressive clinical behavior and frequent metastasis [10, 16]. To investigate the role of CCN6 in breast cancer plasticity, we employed MDA-MB-231 and MDA-MB-436 cells. These cells exhibit a spindle cell morphology and have been recently found to cluster in the mesenchymal stem-like subtype of triple negative breast cancer [25, 26]. Similar to human aggressive breast carcinomas, MDA-MB-231 and -436 cells exhibit low CCN6 protein (Supplementary Figure 1A). Ectopic lentivirus-mediated CCN6 expression was sufficient to induce a morphologic mesenchymal-to-epithelial transition (MET) and to reduce invasion of MDA-MB-231 and -436 cells when compared to controls (Figure 1A-1C).

We next investigated the consequences of CCN6 overexpression on cell motility, a critical step in metastasis. Random cell motion was quantified using live cell imaging with time-lapse microscopy [27]. CCN6 overexpression in MDA-MB-231 and -436 cells significantly decreased the average cell velocity when compared to controls (Figure 1D). Wound healing assays demonstrated that CCN6 overexpression significantly reduced cell migration compared to controls (Figure 1E and Supplementary Figure 2A). Collectively, these experiments show that CCN6 overexpression promotes an MET and reduces the ability of breast cancer cells to move and invade.

### CCN6 overexpression reduces the number of breast tumor initiating cells (TICs)

**Figure 2:**
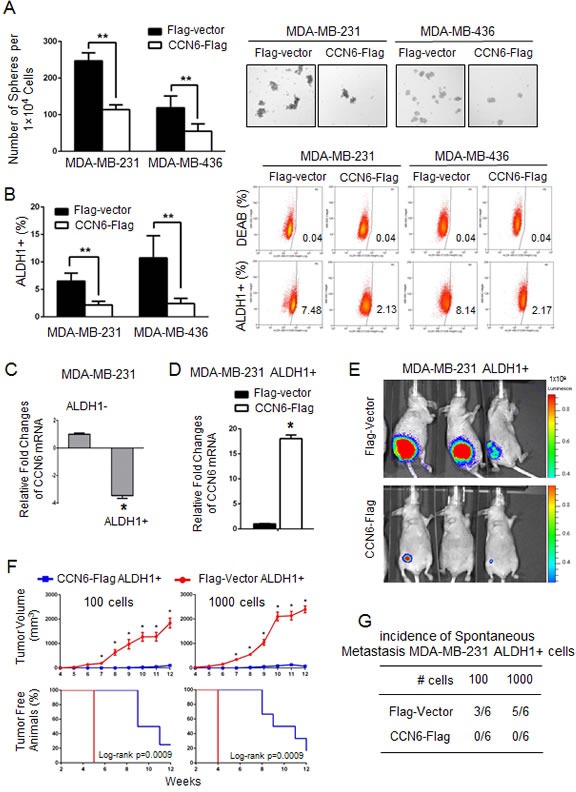
CCN6 overexpression reduces TICs in aggressive breast cancer cells and their tumorigenic ability *in vivo* **A.** CCN6 overexpression in MDA-MB-231 and -436 cells significantly reduces the number of tumorspheres compared to controls. Bars show the average sphere number ±SD per 1×10^4^ plated cells. Representative images of spheres after 7 days in culture (magnification: 200x). **B.** CCN6 overexpression reduces the percentage of ALDH1+ cells using the ALDEFLUOR assay by flow cytometry compared with controls. The percentage of ALDEFLUOR positive cells ± SD is shown. **C.** CCN6 quantitative RT-PCR in MDA-MB-231 ALDH1+ and ALDH1- cells. **D.** CCN6 quantitative RT-PCR in MDA-MB-231 ALDH1+ cells transduced with CCN6-Flag or Flag-Vector. **E.** ALDH1+ MDA-MB-231 cells transduced with CCN6-Flag or with Flag-Vector were injected into the cleared mammary fat pads of NOD/SCID mice (100 and 1000 cells, 6 mice per condition). Representative images of mice injected with 100 cells analyzed at week 11 using luciferase bioluminescence imaging. **F.** *Top*, CCN6 overexpression significantly reduces the volume of tumors formed by MDA-MB-231 ALDH1+ cells compared with controls. Average tumor volume ± SEM for weeks 4-12 post injection for all conditions (mixed regression model, *p < 0.05). *Bottom*, Kaplan Meier plot shows that CCN6 overexpression significantly increases the time to tumor initiation in MDA-MB-231 ALDH1+ cells compared with controls (log-rank *p* = 0.0009 for both conditions). **G.** Table shows the number of mice with metastasis/total number of mice in each group (*n* = 6 mice/group). All metastasis were to the lungs, except one to the soft tissues adjacent to the vertebral column in a Flag-Vector mouse 1,000 cell group. Metastases were diagnosed by histopathology analyses of paraffin-embedded sections.

To test the previously unexplored role of CCN6 in the regulation of breast TICs, we performed mammosphere assays, based on the property of TICs to survive in non-adherent, serum-free culture conditions [28]. CCN6 overexpression in MDA-MB-231 and -436 cells reduced sphere numbers compared with controls (Figure 2A). To identify TICs, we also used the positive activity of aldehyde dehydrogenase 1 (ALDH1) measured by the ALDEFLUOR assay [29]. CCN6 overexpression in MDA-MB-231 and -436 cells significantly reduced the percentage of ALDH1+ cells compared with controls (Figure 2B). Extending these observations to human breast cancer, CCN6 overexpression decreased sphere numbers and reduced the ALDH1+ populations in primary cancer cells derived from a patient with TN invasive carcinoma (Supplementary Figure 1C-1D).

Studies have demonstrated that ALDH1+ breast cancer cells have tumor initiating abilities when injected in the clear fat pads of immunocompromised mice [29]. In MDA-MB-231 cells, *CCN6* mRNA expression was lower in the ALDH1+ population *vs*. the ALDH1- population, indicating that CCN6 is expressed at lower levels in the TICs compared to non-TICs (Figure 2C). To test the hypothesis that CCN6 overexpression in the TICs reduces their tumorigenicity, we injected limiting dilutions of ALDH1+ MDA-MB-231 cells overexpressing CCN6-Flag or Flag-Vector into the cleared mammary fat pads of NOD/SCID mice. CCN6 overexpression significantly delayed tumor onset, decreased tumor volume, and blocked distant metastasis of ALDH1+ MDA-MB-231 cells compared with controls (Kaplan-Meier, log-rank *P* < 0.05; Figure 2D-2G and Supplementary Figure 2B). Collectively, these data show that ectopic CCN6 overexpression in breast cancer cells is sufficient to reduce breast TICs, and that overexpression of CCN6 in the TIC population reduces their tumorigenic and metastatic abilities *in vivo*.

### A CCN6/Slug Axis Regulates MET and TICs in breast cancer cells

**Figure 3:**
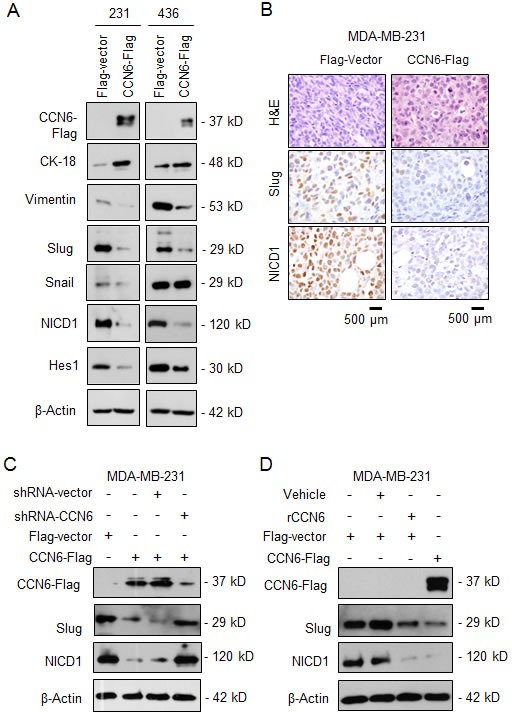
CCN6 regulates the expression of Slug and Notch1 signaling pathway **A.** Immunoblots of MDA-MB-231 and -436 cells transduced with CCN6-Flag or Flag-Vector probed with the indicated antibodies. **B.** Representative images of mammary xenografts derived from MDA-MB-231 transduced with CCN6-Flag and Flag-Vector subjected to immunohistochemistry for the indicated proteins (600x magnification). **C.** Immunoblot of CCN6-Flag and Vector-Flag MDA-MB-231 cells treated with lentivirus containing CCN6 short hairpin (shRNA) or scrambled controls. **D.** Immunoblot of MDA-MB-231 cells treated with recombinant CCN6 (rCCN6) protein (200 ng/mL) or vehicle.

Based on the effect of CCN6 overexpression on MET, we investigated the levels of EMT-TFs by quantitative RT-PCR. Ectopic CCN6 overexpression significantly reduced *Slug* mRNA compared to other EMT-TFs in MDA-MB-231 and -436 cells (Supplementary Figure 2C). Concordantly, CCN6 overexpression induced a protein expression profile of MET with downregulation of Slug and Vimentin, and upregulation of Cytokeratin -18 (Figure 3A). While CCN6 reduced Snail in MDA-MB-231 cells, this was not observed in MDA-MB-436 cells. CCN6 overexpression reduced the intracellular activated form of Notch1, NICD1, which plays important roles in cell plasticity and TIC regulation [30-32] (Figure 3A). The CCN6-dependent downregulation of *Slug* and *NOTCH1* mRNA was detected in the ALDH1+ population compared to controls (Supplementary Figure 2D). *In vivo,* mammary xenografts of MDA-MB-231 cells overexpressing CCN6 exhibited reduced Slug and NICD1 compared to controls (Figure 3B). Validating the specificity of the results, lentivirus-mediated CCN6 shRNA knockdown (KD) efficiently rescued the reduced Slug and NICD1 levels due to CCN6 overexpression (Figure 3C). Treatment with recombinant CCN6 protein was sufficient to reduce Slug and NICD1 levels compared to vehicle treated MDA-MB-231 cells (Figure 3D).

**Figure 4:**
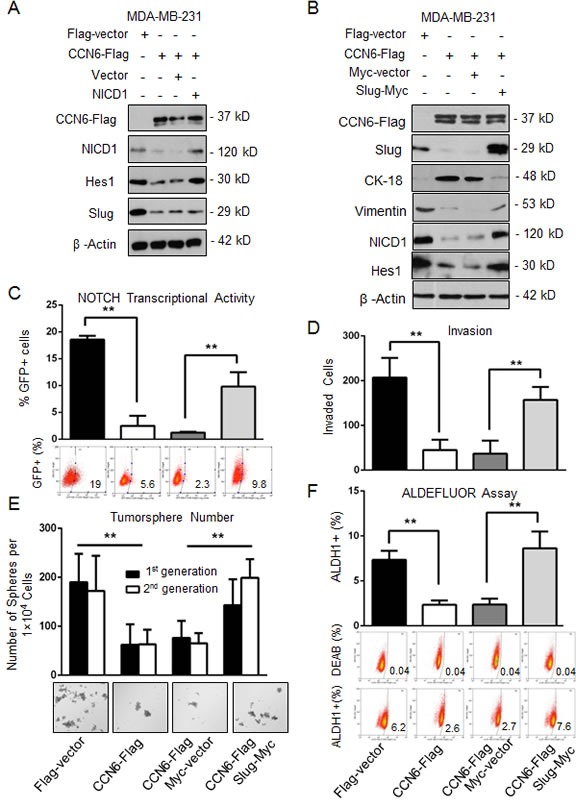
CCN6-dependent reduction of TICs requires Slug downregulation **A.** Immunoblot of MDA-MB-231 CCN6-Flag and Flag-Vector cells transiently transduced with constitutively active NICD1 in a retrovirus vector or vector control [65]. **B.** Immunoblot of MDA-MB-231 CCN6-Flag and Flag-vector transduced with a Slug-Myc construct in a lentiviral vector or control. **C.** Cells described in B were subjected to a GFP-Notch promoter reporter assay. Percentages of GFP-expressing cells ± SD. **D.** Matrigel invasion assay of cells in B. **E.** Tumorsphere formation assays of cells in B. Shown is the average number of tumorspheres per 1 × 10^4^ plated cells in the first and second generations ± SD. Representative images of tumorspheres after 14 days (200x magnification). **F.** ALDEFLUOR assay by flow cytometry of cells in B. *P < 0.05, ** *P <* 0.005, Student's *t* test.

To investigate the mechanistic underpinnings of the observed link between CCN6, Slug, and Notch1 pathway activation we reconstituted Slug or Notch1 expression in CCN6 overexpressing MDA-MB-231 and -436 cells. Whereas ectopic Notch1 overexpression had no effect on Slug protein levels (Figure 4A), Slug overexpression was sufficient to rescue NICD1 and Hes1 proteins and Notch1 transcriptional activity in both cells (Figure 4B and 4C, and Supplementary Figure 3A and 3B). Functionally, Slug overexpression effectively rescued the CCN6-mediated decrease in invasion, the percentage of ALDH1+ cells, and the number of primary and secondary tumorspheres in MDA-MB-231 and -436 cells compared to controls (Figure 4D-4F, and Supplementary Figure 3C-3E). Taken together, these data indicate that Slug is required for CCN6-mediated Notch1 signaling, MET and TIC regulation.

### A conserved TSP1 domain of CCN6 protein regulates Notch1 transcriptional activity, MET, and TICs

**Figure 5:**
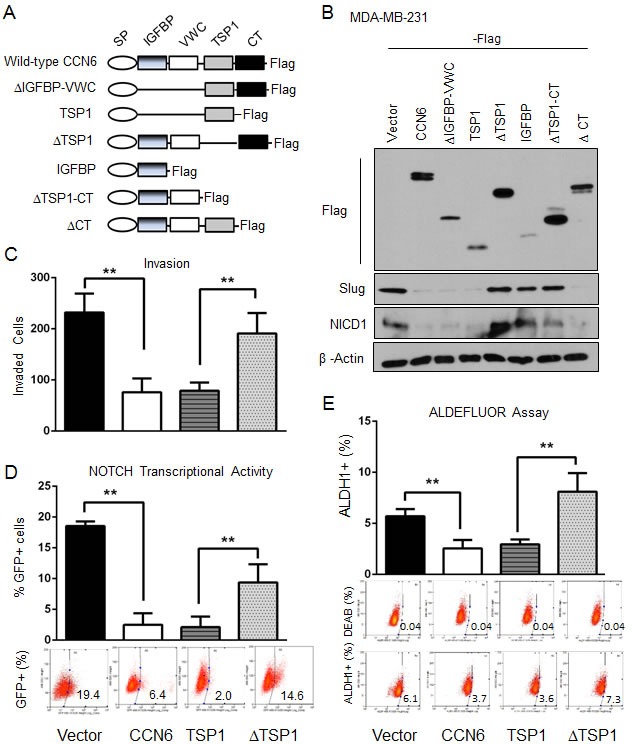
The TSP1 domain of CCN6 is required for CCN6-mediated functions **A.**Schematic diagram of CCN6 and the generated 6 truncated mutants with C-terminal Flag-tag. CCN6 protein contains four domains: IGFBP, VWC, TSP1, and CT with an N-terminal signal peptide (SP). **B.** Immunoblot of MDA-MB-231 cells stably transduced with CCN6 and CCN6 truncated mutants probed with anti-Flag, anti-NICD1, anti-Slug, and anti-β-actin. **C.** Matrigel invasion assay of MDA-MB-231 cells expressing ectopic CCN6, a deletion mutant containing only the TSP1 domain (TSP1), a deletion mutant lacking the TSP1 domain (ΔTSP1) or control. **D.** GFP-NOTCH promoter reporter assay of cells in C. Percentages of GFP-expressing cells ± SD E. ALDEFLUOR assay of cells in C. The percentage of ALDEFLUOR positive cells ± SD is shown. *P < 0.05, **P < 0.005, two-tailed Student's *t* test.

The functions of the conserved motifs of the CCN6 protein are largely unknown. We generated a series of Flag-tagged CCN6 deletion mutants involving the 4 conserved CCN6 domains (Figure 5A). The mutants were developed in lentiviral vectors and expressed in MDA-MB-231 and -436 breast cancer cells (Figure 5B and Supplementary Figure 4A). Ectopic expression of wild-type CCN6 and deletion mutants containing an intact TSP1 domain (ΔIGFBP-WVC, TSP1, and ΔCT) led to Slug and NICD1 downregulation. In contrast, deletion mutants lacking the TSP1 domain (ΔTSP1, IGFBP, and ΔTSP1-CT) were unable to reduce Slug and NICD1 expression levels compared to wild type CCN6, indicating that the TSP1 domain is required for Slug and Notch1 regulation (Figure 5B and Supplementary Figure 4A). Functionally, deletion of the TSP1 domain (ΔTSP1) abrogated the ability of CCN6 to reduce Notch transcriptional activity, cell invasion, the number of ALDH1+ cells, and the number of tumorspheres formed by MDA-MB-231 and -436 breast cancer cells (Figure 5C-5E, and Supplementary Figure 4B-4F). These data demonstrate that the conserved TSP1 domain of CCN6 is crucial to downregulate Slug/Notch1 axis and for CCN6 mediated regulation of MET and TICs in breast cancer cells.

### CCN6 protein is inversely correlated with NICD1 expression in human invasive breast carcinomas

**Figure 6:**
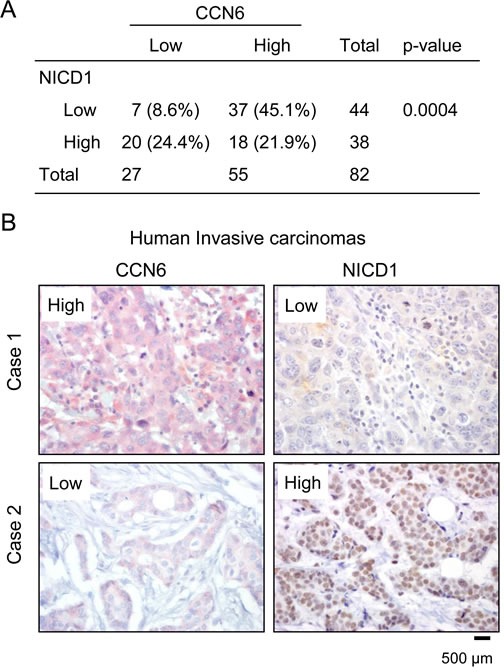
CCN6 expression is inversely associated with NICD1 in human breast cancer tissues **A.** Distribution of CCN6 and NICD1 expression in the patient cohort (*n* = 82). We discovered a significant association between CCN6 and NICD1 proteins, Chi Square test, *p* = 0.0004. **B.** Human breast cancer tissue samples immunostained for CCN6 (red) and NICD (brown). Case 1 is a representative invasive carcinoma with high CCN6 and low NICD1 expression; Case 2 is an invasive carcinoma with low CCN6 and high NICD1 levels.

To assess the relevance of our *in vitro* and animal studies to human breast cancer, we simultaneously investigated the expression of CCN6 and NICD1 proteins in 82 primary invasive carcinoma tissue samples arrayed in a tissue microarray [33]. Immunohistochemical analysis showed that when present, CCN6 protein was predominantly cytoplasmic and that NICD1 protein was localized to the nuclei of breast cancer cells (Figure 6B). CCN6 and NICD1 were scored as high when over 10% of the cancer cells showed moderate or strong staining and were scored as low when staining was present in less than 10% of tumor cells [10, 34, 35]. We found a novel significant association between CCN6 and NICD1 protein expression in invasive carcinomas of the breast. Of the 82 tumors, 57 (69.5%) exhibited reciprocal expression of CCN6 and NICD1 proteins (45.1% had high CCN6 and low NICD1, and 24.4% had low CCN6 and high NICD1), Chi Square test, *p* = 0.0004. High expression of both proteins was seen in 18 of 82 tumors (21.9%), and low CCN6 and NICD1 expression occurred in 7 of 82 tumors (8.6%) (Figure 6A-6B).

## DISCUSSION

This study reveals that the matricellular CCN6 protein modulates breast cancer cell plasticity and points to a new mechanism by which CCN6 regulates the transition between epithelial and mesenchymal states and breast cancer initiating cells. This mechanism is mediated by the TSP1 domain of CCN6 and implicates downregulation of Slug and inhibition of Notch1 signaling pathway.

Extensive studies by many laboratories have sought to unravel the molecular basis of the tumor initiating events and the role of the EMT process on tumor initiation [19, 36-38]. While the contribution of the tumor microenvironment to the initiation and progression of breast and other malignancies is established [1-3], the specific function of matricellular proteins has remained understudied. We present evidence that CCN6 suppresses breast cancer initiation by inhibiting Slug-dependent EMT and TIC programs. Remarkably, ectopic CCN6 overexpression is sufficient to promote an MET and reduce the number of TICs and their tumorigenic and metastatic ability *in vivo*.

Our group has previously reported that CCN6 loss is associated with a highly metastatic form of invasive breast carcinoma, termed inflammatory breast cancer, as well as with non-inflammatory invasive breast cancers with lymph node metastasis [10, 16]. We have found that CCN6 knockdown in non-tumorigenic breast cells induced EMT and increased invasion [10]. However, an effect of CCN6 on TICs has not been previously considered. Data presented here show that CCN6 overexpression reduced the number TICs in TN breast cancer cells. CCN6 overexpression in the breast TIC population significantly delayed tumor development, reduced tumor volume, and blocked the development of spontaneous metastasis. These functions of CCN6 may explain at least in part the enhanced metastasis observed in inflammatory breast cancer and in invasive carcinomas with low or absent CCN6 expression.

We have delineated a molecular pathway through which CCN6 regulates EMT-MET transitions and TICs in breast cancer. Through a combination of knockdown and rescue strategies in aggressive mesenchymal-like breast cancer cells, we demonstrate that ectopic CCN6 overexpression or treatment with recombinant CCN6 downregulates Slug and is sufficient to reduce Notch1 signaling and transcriptional activity, reverse EMT, and reduce the number of TICs. Studies have reported that Notch1 directly binds to the Slug promoter to enhance transcription and induce EMT [39, 40]; however, whether Slug regulates Notch1 and the relationship of these pathways to CCN6 was previously unknown. Through this study, the association and mechanistic link between CCN6, Slug, and Notch1 was validated *in vivo* and *in vitro*.

Our data shed new light on the regulation of Slug during breast tumorigenesis. Slug, a member of the Snail family of zinc finger transcriptional repressors, plays an important role during mammary epithelial cell differentiation [41]. In addition, recent work has demonstrated that Slug promotes mammary stem cell state and regulates normal luminal and basal cells in the breast [22, 42]. Slug plays complex roles during breast tumorigenesis including induction of EMT, differentiation, and regulation of tumor initiating cells [41]. In human breast cancer tissues, Slug is frequently overexpressed in BRCA1-mutated and in basal type tumors [43, 44], and Slug levels correlate with increased metastatic potential and tumor grade [43]. Our data pinpoint CCN6 as a novel regulator of Slug in breast cancer progression.

The mechanism by which CCN6 regulates epithelial and mesenchymal transitions appears to differ in normal cells and in cancer cells. CCN6 overexpression in breast cancer cells resulted in Slug downregulation and MET, which was completely reversed upon Slug overexpression, indicating a central role for Slug in this process. Interestingly, our laboratory has previously reported that CCN6 knockdown in non-tumorigenic breast cells induces a spindle and invasive phenotype with up regulation of the EMT-TFs Snail and ZEB1 [10, 23]. These data are in concordance with a body of literature showing that Snail, Slug and ZEB1 may have different roles in the regulation of cell plasticity and stemness in normal cells and cancer cells [22, 41, 45-47]. Taken together, our results demonstrate that CCN6 regulates the expression of EMT-TFs in a context and cell type-specific manner, and suggest that CCN6 is a powerful regulator of breast cell plasticity.

CCN proteins contain four conserved domains including an insulin-like growth factor-binding protein (IGFBP) domain, the von Willebrand factor C (WVC) domain, a thrombospondin type I repeat (TSP1) domain, and a carboxy-terminal domain [4, 9, 48]. The TSP1 domain of CCN proteins consists of 55 amino acids including 6 conserved cysteine residues and a conserved CSxTCG motif, which in CCN6 is CSRTCG [49, 50] While the function of the TSP1 domain of CCN proteins in normal cells and in cancer is largely unknown, it has been reported to modulate cell adhesion, migration, and proliferation in a cell type and context specific manner [49-53]. Here, we show that an intact TSP1 domain is essential for CCN6 regulatory effects on Slug/Notch1 signaling, MET, invasion, and TICs. The important role for the TSP1 domain in mediating CCN6 functions in breast tumorigenesis underscores our previous study demonstrating that human metaplastic carcinomas carry a frame shift mutation within the TSP1 domain sequence of *CCN6* [54].

While our studies have demonstrated that CCN6 exerts tumor suppressor functions in breast cancer [10, 11, 23, 24, 55, 56], CCN6 may promote tumorigenesis in other organs. CCN6 increased migration of chondrosarcoma cells, which was prevented by anti-αvβ3 and αvβ5 integrin monoclonal antibodies, mitogen-activated protein kinase (MEK) inhibitors [57]. Downregulation of CCN6 using siRNAs reduced cell proliferation and induced apoptosis in bladder cancer cells *in vitro* [58]. Recently, Fang et al showed that silencing CCN6 in gastric carcinoma cells reduces proliferation, migration and invasion *in vitro* at least in part by suppressing Wnt/β-catenin signaling [59]. Our lab has reported that CCN6 downregulation reduces E-cadherin expression in human mammary epithelial cells by recruiting Snail1 and Zeb1 to the E-cadherin promoter [10]. Furthermore, Warman and colleagues found that CCN6 is able to inhibit Wnt signaling in zebrafish and in HEK293T cells [60]. While the molecular basis for the context-dependent functions of CCN proteins is unknown [9, 15, 61-63], it has been proposed that the specific CCN protein motifs have different binding partners depending on the cellular context, and that truncated forms of CCN proteins may exert different biological functions [64].

In conclusion, our results show a previously undescribed function of CCN6 during breast tumorigenesis. We have identified that CCN6 overexpression leads to MET and reduces TICs in aggressive mesenchymal-like breast cancer cells. Our results enable us to pinpoint one novel mechanism by which CCN6 regulates breast cancer cell plasticity implicating a new Slug/Notch1 signaling axis. In view of our results and based on the profound effects of CCN6 overexpression on tumorigenesis and metastasis, we propose that modulation of CCN6 levels may be a potential strategy to prevent or halt breast cancer development.

## MATERIALS AND METHODS

### Cell culture and targeting vectors

MDA-MB-231 and MDA-MB-436 cells were purchased from the American Type Culture Collection and grown under recommended conditions. The construction of CCN6-Flag and its truncated mutants were performed as reported [24]. Human Slug cDNA was amplified by PCR and subcloned into a lentiviral pLentiLox-RSV-puro vector with the Myc-tag at its C-terminus. To reconstitute NOTCH1 intracellular domain (NICD1) expression, MDA-MB-231 cells expressing CCN6-Flag were transiently transduced with constitutively active NICD1 in a retrovirus vector [65]. All viruses were packaged at the University of Michigan Vector Core. Transductions were carried out as reported [24]. Recombinant CCN6 protein (rCCN6) (200 ng/mL) was purchased from PeproTech, and used as in our previous studies [55]. A pGreenFire1-NOTCH plasmid that expresses a destabilized copGFP reporter and firefly luciferase under the control of four NOTCH transcriptional response elements and a minimal CMV promoter (System Bioscience, #TR020PA-1) was transiently expressed in a lentiviral vector for the NOTCH transcriptional activity reporter assays [34].

### Immunoblots

For MDA-MB-231 and -436 breast cancer cells, Western blot analyses were carried out with 100μg of whole cell extract derived as previously reported [66]. Primary antibodies used include: CCN6 (Santa Cruz Biotechnology, #SC-25443), Notch1 (Cell Signaling, #3608); Notch1 (Santa Cruz Biotechnology, #SC-32745); Hes1 (Cell Signaling, #11988), Zeb1 (Santa Cruz Biotechnology, #SC-25388); Cytokeratin18 (Abcam, #AB32118); FlagM2 (Sigma-Aldrich, #A8592); Vimentin (Abcam, #AB16700); Slug (Cell Signaling, #9585); Snail1 (Cell Signaling, #3879); Myc-Tag (Cell Signaling, #2276); β-Actin (Santa Cruz Biotechnology, #SC-47778).

### RNA isolation and quantitative RT-PCR

Total RNA wa isolated using RNeasy Mini Kit (Qiagen, #74104) and 1μg of total RNA were reverse transcribed to complementary DNA (cDNA) using Applied Biosystems High-Capacity cDNA Reverse Transcription Kit (Applied Biosystems, #4368814). Quantitative real-time RT-PCR (qRT-PCR) was performed with Applied Biosystems StepOnePlus RT-PCR System available in the Michigan MicroArray Core using the Power SYBR Green PCR Master Mix (Applied Biosystems, #4367659). A housekeeping gene (GAPDH) was used as an internal standard. The primers used in this study are described in Supplementary Table 1.

### Tumorsphere assays

Single cell dissociation for mammosphere formation assays was performed following established protocols with MDA-MB-231 and MDA-MB-436 cells plated at a density of 1×10 ^4^ cells/mL [28]. Tumorspheres were cultured in MammoCult Human Basal Medium with added Proliferation Supplement (StemCell Technologies, #05621 & #05622) on Costar Ultra Low Attachment tissue culture plates (Corning, #3471). At the end of seven days, for both primary and secondary generations, mammosphere sizes and numbers were determined using a Leica inverted microscope. Size was measured as the widest diameter with the scale bar. All experiments were performed in triplicate.

### Flow cytometry

ALDEFLUOR assay was used for detection of the stem cell population (StemCell Technologies, #01700) following the manufacturer's instructions. For NOTCH reporter assays, 1×10^6^ cells transduced with the NOTCH reporter lentivirus (described above), were subjected to flow cytometry to determine the percentage of GFP positive cells. All flow cytometry analyses were completed using the University of Michigan Flow Cytometry Core in triplicate.

### Invasion assay

*In vitro* invasion was measured using 24-well matrigel-coated invasion chambers (BD Biosciences, Bedford, MA) according to the manufacturer's instructions, in triplicate. Invasive cells on lower sides of chambers were stained with 0.05% crystal violet, air-dried, photographed, and then counted under a microscope.

### Wound healing assay

Cells were cultured in a 6-well-plate for 24~36h until forming confluent monolayer. To achieve it, total 1×10^6^ of MDA-MB-231 cells were seeded in 2ml complete medium/well (concentration: 5×10^5^/ml), so did for MDA-MB-436 cells with total 5×10^5^ cells in 2ml complete medium/well (concentration at 2.5×10^5^ cells/ml). When it reached confluent monolayer, a linear scratch was created using a 200 μl sterile pipette tip. Wounded monolayers were washed with PBS for 3 times to remove detached cells and debris, and the complete medium was then added. Pictures were taken at 3 different time point: 0h, 12h and 24h, by using phase contrast microscope with magnification at 100×. The scratched area without cell growth was measured using MTrackJ/ImageJ software.

### Migration assays

Random motion cell motility assays were completed as previously described [27]. Briefly, cells were plated on collagen-coated chambered coverslips at low density attaching overnight. Next day, cells were imaged every 10 minutes at 37°C for 24 hours using the DeltaVision RT Live Cell Imaging System (Applied Precision, GE Healthcare) equipped with a UPlanAo 20X/0.7 NA lens at the University of Michigan Microscopy and Image analysis Laboratory. DIC images were acquired using SoftWoRx 3.5.1 software, and cell movements were quantified using MTrackJ/ImageJ software.

### Immunohistochemistry

A high-density tissue microarray (TMA) containing triplicate samples of 82 human primary invasive breast carcinomas developed and previously characterized by our group were employed [33]. The TMAs were subjected to immunohistochemistry to detect CCN6 and NICD1 proteins. Five micron-thick paraffin-embedded sections were de-paraffinized in xylene and rehydrated through graded alcohols to water. Heat Induced Epitope Retrieval (HIER) was performed in the Decloaking Chamber (Biocare Medical) with Target Retrieval, pH 6.0 (DakoCytomation). Slides were incubated in 3% hydrogen peroxide for 5 minutes to quench endogenous peroxidases. Anti-CCN6 (Orbigen, 1:300) and anti-NOTCH1/ N-Terminus (Fisher, 1:600) were incubated with the TMAs for 1.5 hours at room temperature. Antibodies were detected with Envision^+^ HRP Labeled Polymer (DakoCytomation) for 30 minutes at room temperature. HRP staining was visualized with the DAB^+^ Kit (DakoCytomation). Negative control slides were run. Slides were counterstained in hematoxylin, blued in running tap water, dehydrated through graded alcohols, cleared in xylene and then mounted with Permount. Expression of CCN6 and NICD1 was evaluated as either low or high based on intensity of staining and percentage of staining cells [10, 34, 35].

### *In vivo* studies

The ALDH1+ cell population isolated using ALDEFLUOR assay from firefly luciferase expressing MDA-MB-231 cells expressing Flag-vector or CCN6-Flag were injected orthotopically into the right inguinal cleared fat pad of NOD/SCID mice (100 and 1,000 cells per mouse, 6 mice per group). Mice were euthanized when primary tumor volumes reached 2.0 cm^3^. Metastases were monitored by firefly luciferase bioluminescence imaging by BLI.

### Statistical analyses

Data are expressed as mean ± SD. All experiments were repeated at least 3 times with similar results. The 2-tailed Student's *t* test was performed to determine the probability of statistically significant difference (P values) and recorded in figure legends. Survival curves were calculated using the Kaplan-Meier method. Statistical significance for survival between groups was analyzed by log-rank test. A P value less than 0.05 was considered statistically significant. For all analyses, P values were obtained using Student's *T*-test unless specified otherwise.

## SUPPLEMENTARY MATERIALS FIGURES AND TABLE


