# Supplementary material for: The matricellular protein CCN6 (WISP3) decreases Notch1 and suppresses breast cancer initiating cells
Source: Oncotarget. 2016 Feb 25;7(18):25180–93. doi: 10.18632/oncotarget.7734 (PMC5041896; doi:10.18632/oncotarget.7734)
Supplement: Supplementary file 1 [file oncotarget-07-25180-s001.pdf]

The matricellular protein CCN6 (WISP3) decreases Notch1 and suppresses breast cancer initiating cells

Supplementary Material

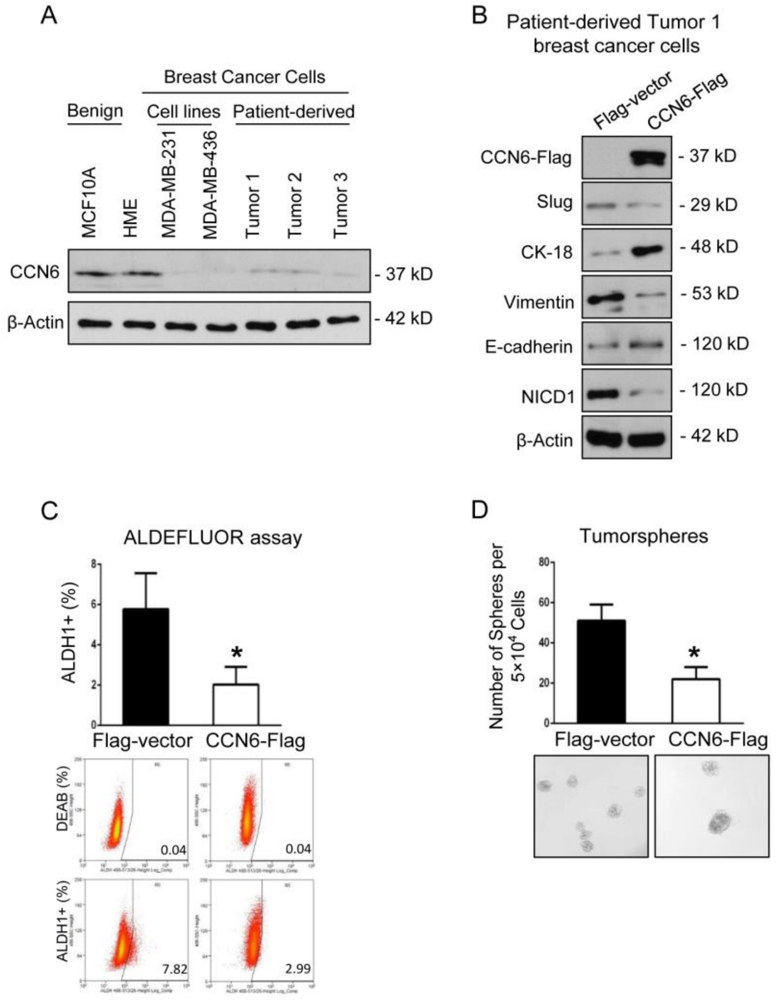

Supplementary Figure 1. CCN6 overexpression in a patient-derived breast cancer cell reduces ALDH1+ cells and the number of tumorspheres. A. Immunoblot for CCN6 protein in a panel of breast cells, as indicated. **B.** Immunoblot of patient derived breast cells transduced with lentivirus containing CCN6-Flag or Vector-Flag. **C.** ALDEFLUOR assay showing that CCN6 overexpression reduces the percentage of ALDH1+ cells compared with controls. The percentage of ALDEFLUOR positive cells ±

SD is shown. **D.** CCN6 overexpression in patient derived breast cancer cells significantly reduces the number of tumorspheres compared to controls. Bars show the average sphere number ±SD per $5x10^4$ plated cells. Representative images of spheres after 7 days in culture (magnification: 200x).

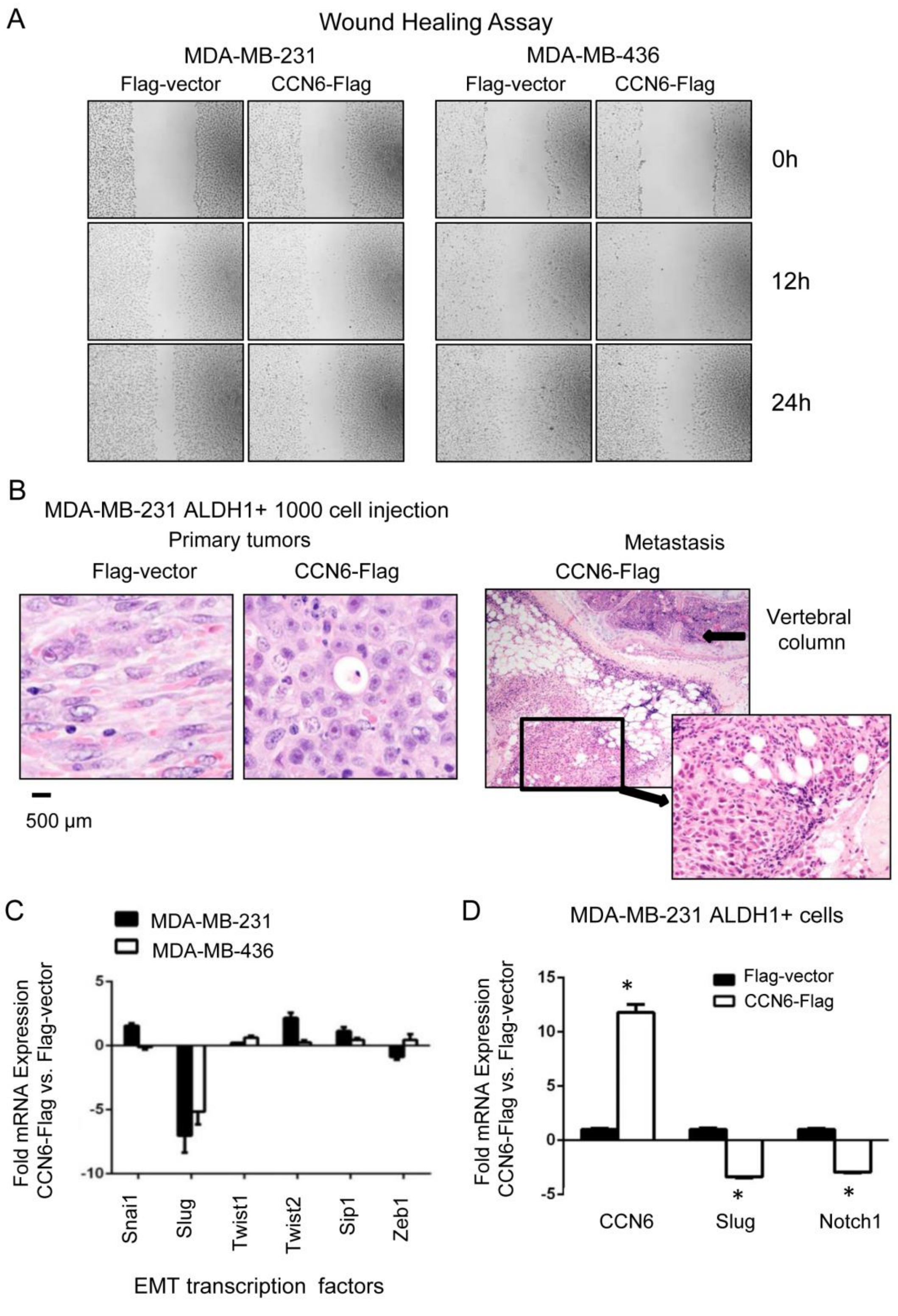

Supplementary Figure 2. A. Representative images of the wound healing assays performed on MDA-MB-231 and -436 cells transduced with Flag-Vector or CCN6-Flag,

the quantification graphs are shown in Fig. 1E. **B**. Representative hematoxylin and eosin (H&E)-stained sections of primary tumors derived from injecting 1000 MDA-MB-231 ALDH1+ cells transduced with CCN6-Flag and Flag-Vector, 600x magnification. Shown is also a picture of a soft tissue metastasis of Vector-Flag transduced cells, 200x magnification. The inset shows a high power of the cancer cells invading the paravertebral adipose tissue, 400x magnification. **C**. Quantitative RT-PCR for EMT-TFs in MDA-MB-231 and -436 cells transduced with CCN6-Flag or Flag-Vector. **D.** Quantitative RT-PCR to determine *CCN6*, *Slug,* and *NOTCH1* mRNA levels in MDA-MB-231 ALDH1+ cells transduced with CCN6-Flag or Flag-Vector.

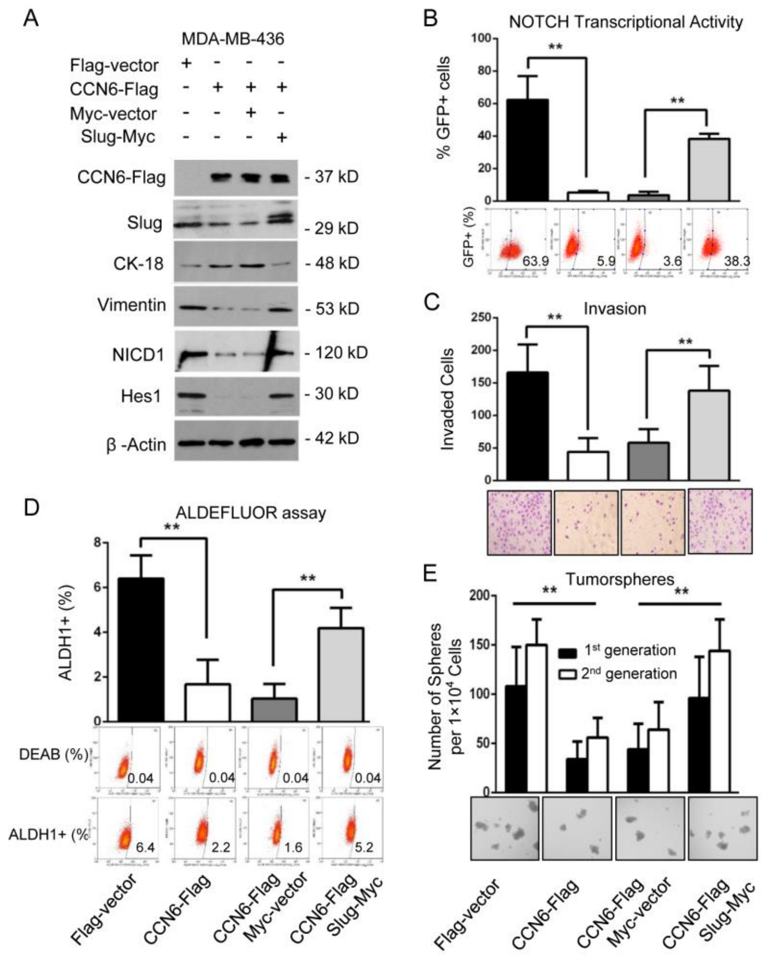

Supplementary Figure 3. CCN6-dependent reduction of TICs in MDA-MB-436 requires downregulation of Slug. A. Immunoblot of MDA-MB-436 cells transduced with lentivirus containing CCN6-Flag and Flag-vector. Slug was rescued using a Myc construct in a lentiviral vector. **B**. GFP-Notch promoter reporter assay at the indicated conditions. Percentages of GFP-expressing cells ± SD. **C.** Matrigel invasion assay of cells in A. **D.** ALDEFLUOR assay by flow cytometry of cells in A. **E.** Tumorsphere formation assays of cells in A. Shown is the average number of tumorspheres per 1 x 10^4 plated cells in the first and second generation ± SD. Representative images of tumorspheres after 14 days (200x magnification). **P*<0.05, ** *P*<0.005, Student's t test.

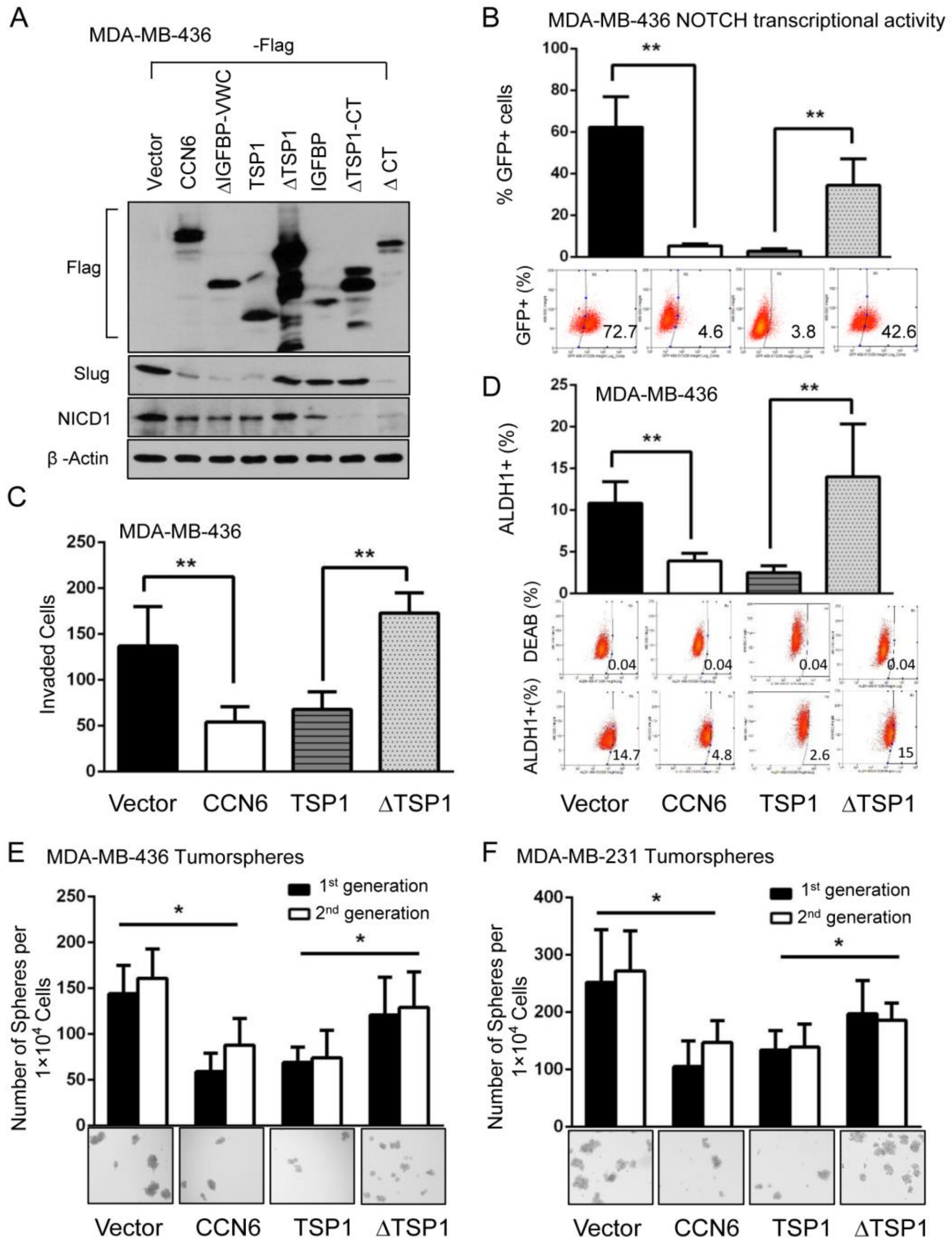

Supplementary Figure 4. The TSP1 domain of CCN6 is required for CCN6-mediated functions in MDA-MB-436 cells. A. Immunoblot of MDA-MB-436 cells stably transduced with CCN6 and CCN6 truncated mutants probed with anti-Flag, anti-NICD1, anti-Slug, and anti-β-actin. **B.** GFP-NOTCH promoter reporter assay of MDA-MB-231 cells expressing ectopic CCN6, a deletion mutant containing only the TSP1

domain (TSP1), a deletion mutant lacking the TSP1 domain (ΔTSP1) or control cells. Percentages of GFP-expressing cells ± SD. **C.** Matrigel invasion assay of cells in A. **D.** ALDEFLUOR assay of cells in A. The percentage of ALDEFLUOR positive cells ± SD is shown. **P*<0.05, ***P*<0.005, two-tailed Student's t test. **E-F.** Tumorsphere formation assays of MDA-MB-231 and MDA-MB-436 cells expressing ectopic CCN6, TSP1, ΔTSP1 or controls. Shown is the average number of tumorspheres per 1 x 10^4 plated cells in the first and second generation ± SD. Representative images of tumorspheres after 14 days (200x magnification). **P*<0.05, ** *P*<0.005, Student's t test.

Supplementary Table 1. Primers used for quantitative RT-PCR

Gene	Orientation	Sequences (5' to 3')
ccn6	Forward	acagggcactggaccattag
	Reverse	ggttgcttggcacagatttt
notch1	Forward	tacaagtgcaactgcctgct
	Reverse	cagaacgcactcgttgatgt
snail	Forward	gcgagctgcaggactctaat
	Reverse	cccactgtcctcatctgaca
slug	Forward	ttggagcagtttttgcactg
	Reverse	ccctcaaagacagcctgaac
twist1	Forward	ggagtccgcagtcttacgag
	Reverse	tggaggacctggtagaggaa
twist2	Forward	agcaagaagtcgagcgaaga
	Reverse	cagcttgagcgtctggatct
sip1	Forward	aatggcaacagcaacaagtg
	Reverse	ccccgtcagcacataacttt
zeb1	Forward	gcacaaccaagtgcagaaga
	Reverse	catttgcagattgaggctga
gapdh	Forward	gcaccaccaactgcttagca
	Reverse	gtcttctgggtggcagtgatg
